# Treatment practices and outcomes of chest indrawing pneumonia in children aged 2–59 months in primary health facilities of Kamuli District, Eastern Uganda

**DOI:** 10.7189/jogh.16.04021

**Published:** 2026-01-23

**Authors:** Ezekiel Mupere, Marble Nasasira, Lilian Tabwenda, Harriet M Babikako, Lorna Muhirwe, Jesca Nsungwa Sabiiti, Shamim Ahmad Qazi, Yasir Bin Nisar

**Affiliations:** 1 Department of Paediatrics and Child Health School of Medicine College of Health Sciences, Makerere University, Kampala, Uganda; 2 Child and Family Foundation Uganda, Kampala, Uganda; 3 Child Health and Development Centre School of Medicine College of Health Sciences, Makerere University, Kampala, Uganda; 4 OneWorld Health, Kampala, Uganda; 5 Reproductive Maternal and Child Health, Ministry of Health, Kampala, Uganda; 6 Independent Child and Neonatal Health Consultant, Geneva, Switzerland; 7 Department of Sexual, Reproductive, Maternal, Child and Adolescent Health and Ageing, World Health Organization, Geneva, Switzerland

## Abstract

**Background:**

The World Health Organization revised its pneumonia guideline for managing children with chest indrawing and/or fast breathing, classifying them as 'pneumonia' and treating them with oral antibiotics at home; the Integrated Management of Childhood Illness (IMCI) protocol was revised accordingly. Evidence is needed on outcomes and treatment practices with the application of revised guidelines in programme settings.

**Methods:**

This prospective observational cohort study was conducted in seven selected health centres in Uganda from November 2022 to May 2023. The IMCI-trained health workers identified and enrolled children aged 2–59 months presenting with cough and/or difficult breathing and chest indrawing without any general danger signs. The primary outcome was vital status at day 15 after the initial assessment. Secondary outcomes were prevalence of and adherence to antibiotic use, and hospitalisation. Enrolled children were given age-appropriate treatment.

**Results:**

Of the 316 children who were enrolled, 68.4% (n/N = 216/316) were aged 12–59 months, and 93.7% (n/N = 296/316) had comorbidities, primarily malaria and diarrhoea. All were prescribed oral amoxicillin. Two children were lost to follow-up; thus, we followed 314 children on day 15. In children aged 2–11 months, 93.9% (n/N = 93/99) received a correct prescription compared to 20% (n/N = 43/215) among 12–59-month-olds. Adherence to five days of treatment was reported for 64.3% (n/N = 202/314) of children. According to the mothers' self report, no deaths were reported, 95.2% (n/N = 299/314) were cured; 2.2% (n/N = 7/314) were worse with six of seven hospitalised, and 2.5% (n/N = 8/314) were the same as the condition at time of enrolment. Most children were well-nourished; 3.8% had a weight-for-height (WHZ) z-score<−3, 6.7% had a weight-for-age (WAZ) z-score<−3, and 0.3% had a mid-upper arm circumference (MUAC)<115 mm. At follow-up on day 15, of 16 children hospitalised at any time after enrolment, 10 (62.5%) had recovered and were discharged, while six (37.5%) were still hospitalised. The presence of any severe malnutrition was associated with a 4-fold increased risk of hospitalisation. In contrast, a longer duration of oral amoxicillin treatment was associated with a 66% decrease in risk of hospitalisation during the follow-up period.

**Conclusions:**

Children aged 2–59 months with chest indrawing pneumonia without danger signs can be successfully managed at home with a five-day course of oral amoxicillin, highlighting the importance of the new policy and approach.

**Registration:**

ISRCTN12687253.

Globally, pneumonia is the leading cause of morbidity and mortality in children, contributing to 15% of deaths among children aged 1–59 months [1]. It is equivalent to over 725 000 deaths in 2021 alone [2,3]. Children in sub-Saharan Africa and South Asia disproportionately bear the burden of pneumonia [4], and Uganda is among 15 countries with the highest number of estimated pneumonia-related deaths [5]. Close to 25 children die of pneumonia every day in Uganda [6]. Evidence indicates that increasing antibiotic treatment coverage, a proven and relatively inexpensive intervention, could reduce pneumonia deaths by 51% in 2025 [7]; however, only a small proportion of children with pneumonia actually receive an antibiotic [8].

Management of children with diarrhoea, pneumonia, malaria, measles and/or malnutrition was integrated into the United Nations International Children's Emergency Fund (UNICEF)/World Health Organization (WHO) strategy, Integrated management of childhood illness (IMCI), for health workers in high-risk settings [9,10]. The IMCI recommends using clinical signs for classification, treatment and place-of-treatment decisions for children up to five years of age. The original recommendation for children with a history of cough and/or difficult breathing with chest indrawing, with or without danger signs, was hospitalisation for injectable antibiotics and supportive therapy [9]. However, increasing evidence demonstrated that in resource-limited settings, many children with chest indrawing pneumonia did not receive the recommended inpatient treatment because such treatment was not accessible, acceptable or affordable to many families [11–14]. While improving the availability of and access to hospital-based therapy is imperative, providing effective treatment for children with chest indrawing pneumonia on an outpatient basis would increase access to life-saving care [15–18]. Moreover, in resource-limited settings, caregivers have a positive attitude and good practices towards home management of pneumonia [19].

In 2012, given new evidence, WHO revised the classification of pneumonia such that children with chest wall indrawing, with or without fast breathing, were classified as 'pneumonia' and managed with oral antibiotics at home [20,21]. The WHO updated the IMCI chart booklet in 2014, and many countries, including Uganda, adopted the revised recommendation [22,23]. This new approach is potentially cost-effective, can reduce the number of referrals for hospitalisation and can increase access to treatment, thus leading to better outcomes [24,25]. Concerns have been raised about the applicability of the new guidance in high-mortality areas [26,27], highlighting the need for further evidence in programme settings.Based on this background, we undertook a cohort study to evaluate survival outcomes and treatment practices under the revised IMCI pneumonia protocol among children aged 2–59 months in a programme setting in primary health care facilities in Uganda.

## METHODS

### Study setting

We conducted a prospective cohort study in Kamuli district (Busoga region), with a population of 540 252 [28]. This rural area is mainly representative of Uganda, where subsistence agriculture is a primary livelihood [6,28]. Malaria is endemic, with seasonal peaks from March to May and September to December [6,29]. The study period (November 2022 to May 2023) coincided with a period of reduced malaria risk. Kamuli has a relatively high prevalence of acute respiratory tract infections [6] and is one of the settings where revised IMCI pneumonia guidelines have been implemented.

The study was conducted in seven purposively selected primary care level three health centres (HC3): Bugulumbya, Bulopa, Butansi, Kitayunjwa, Mbulamuti, Nabirumba and Namasagali, each serving a population of approximately 30 000 people. All HC3 clinics operate 24 hours a day in provision of maternal services.

### Participants

Children aged 2–59 months presenting with cough and/or difficult breathing, plus observed chest indrawing, but no general danger signs (convulsions, inability to drink or breastfeed, vomiting everything, lethargic/unconscious), were enrolled. Children not residing within the facility's catchment area and therefore not able to be followed up at home were excluded. Children with associated comorbidities such as malaria were not excluded from the study.

### Initial assessment of patients by the health workers

Before, the health facility in-charge selected the clinical officers and nurses with prior IMCI training to attend a three-day hands-on refresher training. Also, for every 100 households, two community health workers (Village Health Teams (VHTs)) were trained in the Uganda-adapted WHO Integrated Community Case Management (iCCM) training. Their supervisors (Health Assistants) also attended a two-day iCCM orientation. Following the IMCI refresher training, the health facility in-charges in each of the seven selected facilities assigned five staff members who had attended the three-day hands-on refresher training to be responsible for the routine management of sick children.

Between 8:00 *am* and 5:00 *pm*, including weekends and public holidays, the IMCI-trained workers assessed children aged 2–59 months who presented with cough and/or difficult breathing. They also documented clinical information on the IMCI sick child form, including weight, height/length and mid-upper arm circumference (MUAC) measurements, illness classification, treatment, and counselling of the caregiver. The health worker further screened for children 2–59 months old with chest indrawing pneumonia but without stridor in a calm child or any general danger signs and referred them to the study staff. The study staff obtained consent from parents or caregivers, enrolled children in the study and followed up on day 15. The study staff also collected background information from participating caregivers, including maternal educational status and immunisation history, the child's home address and phone contact. More details about this study have been published elsewhere [30].

### Treatment of pneumonia

Enrolled children were provided with age-appropriate medicines, including antibiotics. According to the IMCI Chart booklet, caregivers were instructed on the appropriate use of these medicines at home, advised on the danger signs to watch for, and when to seek further care.

One trained data staff member from the study project was assigned to extract paper-based data into the Research Electronic Data Capture (REDcap^@^), with error checks at the point of entry via automated daily review, and with regular external weekly data quality assurance. These individual case report forms were then stored at the facility in preparation for the follow-up exercise.

### Follow-up

Before leaving the clinic and after consent, each child was assigned to a specific trained VHT member for a follow-up home visit 15 days later. These visits were done under the supervision of the health assistants, who are responsible for community health and for the supervision and mentorship of VHTs.

At the follow-up visit, caregivers were interviewed regarding the child's clinical status, adherence to the medications advised by the health worker and whether any hospitalisation occurred. Anthropometric measurements were taken. Information was collected using a standardised checklist.

### Data analysis

#### Study outcomes

The primary outcome was vital status on day 15 following the initial assessment at the participating facilities. Successfully 'cured' was based on maternal recall, defined as those who are not in the 'same' or the 'worse' condition compared to the time of enrolment and who were not hospitalised at the time of follow-up.

#### Primary outcome

The primary outcome measure of the study was the proportion of children alive on day 15, with 95% confidence intervals (CI).

#### Secondary outcomes

Measures were the proportion (with 95% CI) of children treated correctly, those reported cured, and those who had been hospitalised at any time after recruitment. Correct treatment was defined as children who received medicines according to the health worker's advice. Hospitalisation was defined as any admission between days two and 14 after the initial assessment at the general district hospital or participating facility where possible.

### Sample size

To estimate the survival outcome of sick children treated on an outpatient basis with a precision of ±3%, assuming a 5% risk (based on data from Malawi, personal communication from Humphreys Nsona), α = 0.05, and 5% loss to follow-up [31], the minimum sample size was 310 caregivers and children [30].

### Data management and statistical methods

All data were collected on case report forms and converted to digital format with error checks at the point of entry. Individual child records from initial and follow-up assessments were stored in a participant's study file. All data were double-entered into EpiData 3.1. STATA/IC 16.1 (StataCorp LLC College Station, Texas, USA) statistical software was used for analysis. Participant characteristics, including maternal characteristics and child anthropometric status, were summarised as means ± standard deviation (SD) and medians with interquartile range (IQR) for continuous measures, and proportions for discrete measures. Descriptive statistics were used for patient demographics, IMCI clinical classification and management practices. Adherence to oral amoxicillin therapy was categorised into three groups: five days (10 doses), three to four days (6 to 9 doses) or fewer than three days (less than six doses).

A series of univariate and multivariable logistic regression models were fit to identify factors associated with hospitalisation. Collected sociodemographic and clinical variables known or likely to be associated with hospitalisation were fit into univariate, full and parsimonious multivariable models after testing that the variables fulfilled the assumptions of logistic regression and the outcome variable was binary (yes/no) [32]. Duration of treatment was operationalised as continuous, and the following as categorical variables: age two to 11 months *vs*. 12 to 59 months, male and female gender, up-to-date immunisation status any time based on immunisation card, presence of comorbidity, axillary temperature of ≥ 37.5°C, presence of malaria or diarrhoea, and presence of any severe form of child wasting as measured by MUAC<11.5 cm, weight-for-height or for-length (WHZ) z-score<−3 and weight-for-age (WAZ) z-score<−3. We tested for interactions between variables, particularly age and the duration of treatment or the presence of severe malnutrition. The backward elimination method [33] was used to determine the final parsimonious model. All variables with a *P*-value of <0.05 were considered significant. We used a negative likelihood ratio test to evaluate differences between models and, finally, to assess the effect of confounding; we considered a difference of greater than 10% between the adjusted and unadjusted effect estimates.

### Ethical considerations

Makerere University College of Health Sciences, School of Biomedical Sciences Research Ethics Committee (SBS-2022–168), Uganda National Council for Science and Technology, and the WHO Ethics Review Committee approved the study. Participants gave informed written consent to participate in the study.

## RESULTS

### Baseline characteristics of study participants

Over a 7-month study period (November 2022 to May 2023), 9937 sick children aged 2–59 months from seven primary care facilities were screened, and 4412 (44.4%) presented with of cough/cold or difficult breathing. A health worker classified these children as having cough or cold: 2118/4412 (48.0%), fast breathing: 1445/4412 (32.8%), chest indrawing: 321/4412 (7.3%), severe pneumonia: 23/4412 (0.5%) and either coryza or respiratory tract infection: 505/4412 (11.5%) (**Figure 1**). Parents/caregivers of five of the 321 (1.6%) children with chest indrawing pneumonia did not give consent and thus, these children were not recruited.

The mean (SD) age of study children was 20.4 ± 13.3 months; 68.4% of enrolled children were 12–59 months of age; 52.9% were male, and 72.8% had an axillary temperature of ≥ 37.5°C (**Table 1**). Based on the presence of an immunisation card, on average, 47.9% had an up-to-date immunisation status. Most children were well-nourished; 3.8% had a WHZ z-score<−3, 6.7% had a WAZ z-score<−3, and 0.3% had a MUAC<115 mm. Nearly two-thirds of the caregivers were educated, 24.4% had attained secondary or higher education, and 38% had never attended school. A larger number of children were seen at the Bugulumbya (24.7%) and Buloopa (20.6%) health centres, whereas the Namasagali health centre (2.2%) saw the fewest children.

**Table 1:** Baseline characteristics of enrolled children (n = 316)

Characteristics	Category	Values*
**Child**		
Sex	Female	149 (47.1)
	Male	167 (52.9)
Age in months	Mean (SD)	20.4 ± 13.3
	Median (IQR)	18 (8.5, 30)
Age category	2–11 mo	100 (31.7)
	12–59 mo	216 (68.4)
Up-to-date immunisation based on immunisation card	Yes	136 (47.9)
	No	180 (52.1)
Up-to-date immunisation based on immunisation card (n = 136)	2–11 mo	75 (55.1)
	12–59 mo	61 (44.9)
Temperature categorised	≥ 37.5°C	230 (72.8)
	<37.5°C	86 (27.2)
Temperature	Mean (SD)	37.8 (1.0)
	Median (IQR)	37.7 (37.2, 38.2)
WAZ z-score (n = 314)	Mean (SD)	−0.2 (1.9)
	<−3	21 (6.7)
	≥ −2, −3	20 (6.4)
	>3S	273 (86.9)
WHZ z-score (n = 311)	Mean (SD)	3.6 (15.7)
	<−3	12 (3.8)
	≥ −2, −3	13 (4.1)
	>3	286 (90.5)
HAZ z-score (n = 311)	Mean (SD)	−1.8 (3.5)
	<−3	86 (27.2)
	≥ −2, −3	45 (14.2)
	>3	180 (57.0)
MUAC (n = 275)	<115	1 (0.3)
	≥ 11.5, −<12.5	4 (1.3)
	≥ 12.5	270 (85.4)
	Missing (age <6 mo)	41 (13.0)
**Maternal**		
Ever attended school (n = 314)	Yes	194 (62.0)
	Illiterate/can only sign	120 (38.0)
Mother's education (n = 314)	Primary	117 (37.0)
	Secondary or higher	77 (24.4)
**Health facility**	Bugulumbya	78 (24.7)
	Buloopa	65 (20.6)
	Butansi	45 (14.2)
	Kitayunjwa	37 (11.7)
	Mbulamuti	48 (15.2)
	Nabirumba	36 (11.4)
	Namasagali	7 (2.2)

HAZ – height-for-age z score, IQR – interquartile range, MUAC – mid-upper-arm circumference, SD – standard deviation, WAZ – weight for age Z score, WHZ – weight for height Z score,

*Values are means ± SDs or n (%).

### Pneumonia comorbidity

At baseline, 296/316 (93.7%) children with chest indrawing pneumonia had a co-morbidity (Table S1 in the **Online Supplementary Document**), and most (76.0%) had only one comorbidity. Malaria was the most common (53.8%), followed by diarrhoea (20.1%).

### Health worker treatment practice and adherence at home

All 316 children with chest indrawing pneumonia were prescribed oral amoxicillin by IMCI-trained health workers; two were lost to follow-up. Of the 314 children with chest indrawing pneumonia, health workers prescribed the correct strength and dose of oral amoxicillin in 136/314 patients (43.3%) (Table S2 in the **Online Supplementary Document**). Among the 136 patients with a correct prescription, 93 (68.4%) were aged 2–11 months, while 43 (31.6%) were 12–59 months old. Adherence data for prescribed oral amoxicillin were analysed, regardless of whether a correct prescription was made, nearly two-thirds of children (202/312, 64.7%) adhered to the full five days of the advised treatment (Table S2 in the **Online Supplementary Document**).

### Clinical outcomes

All children who were treated on an outpatient basis with oral amoxicillin survived. According to mothers, of 314, 299 (95.2%; 95% CI = 0.922–0.971) were reported as improved/cured; 7 (2.2%; 95% CI = 0.011–0.046) were reported to be worse with 6 of 7 hospitalised, while 8 (2.5%; 95% CI = 0.013–0.050) were reported as the same compared to the condition at the time of enrolment (**Table 2**).

**Table 2:** Outcome status of enrolled children

Item		Frequency (%) n = 314	95% confidence interval
**Outcome status (as reported by caregiver)**			
Improved		299 (95.2)	0.92, 0.97
Same		8 (2.5)	0.01, 0.05
Worse		7 (2.2)	0.01, 0.05
Death		0 (0.0)	0.01, 0.01
**Ever hospitalised before follow-up on day 15**		16 (5.1)	0.03, 0.08
Hospitalised at the time of the follow-up visit		6 (38.0)	0.15, 0.65
Ever hospitalised but discharged at the time of the follow-up visit		10 (62.0)	0.35, 0.85
**Duration of hospitalisation (n = 16)**			
≤3 d		5 (31.3)	0.11, 0.59
4 d ≥5 d		3 (18.8)	0.04, 0.46
>5 d		2 (12.5)	0.02, 0.38
Still admitted		6 (37.5)	0.15, 0.65
**Duration between diagnosis and admission (n = 16)**			
≤ 3 d		11(68.8)	0.41, 0.89
4 d ≥ 5 d		2 (12.5)	0.02, 0.38
After 5 d		3 (18.7)	0.04, 0.46

Of the 16 children (16/314, 5.1%; 95% CI = 0.918–0.969) who were hospitalised at any time after enrolment, six (6/16, 37.5%; 95% CI = 0.184–0.615) were still hospitalised by the day of the follow-up visit. Ten (10/16, 62.5%; 95% CI = 0.385–0.816) had been discharged as recovered. Eleven of the hospitalised children (11/16, 68.8%) were admitted within three days of initiating treatment on oral amoxicillin.

Eleven (11/16, 68.8%) of the hospitalised children had malaria, confirmed by a malaria rapid diagnostic test (MRDT) at the initial assessment. Of the eleven hospitalised children with confirmed malaria, eight (50%) received an analgesic, four (25%) were continued on oral amoxicillin, and three (18.8%) received an injectable antibiotic (data not shown).

The presence of any severe malnutrition was associated with a 4-fold increased risk of hospitalisation, and a longer duration of oral amoxicillin treatment was associated with a 66% decrease in the risk of hospitalisation during the follow-up period (**Table 3**).

**Table 3:** Factors associated with hospitalisation identified by logistic regression

Variable	Category	Value*		cOR (95% CI)	aOR (95% CI)
		**Hospitalised (n = 16)**	**Not hospitalised (n = 298)**		
Age in months	2 to 11 mo	3 (18.8)	96 (32.2)		
	12 to 59 mo	13 (81.2)	202 (67.8)	2.06 (0.57, 7.40)	4.66 (0.92, 23.55)
Duration of treatment in days treatment	Mean (SD)	4.2 (1.3)	4.8 (1.2)	0.39 (0.20, 0.73)	0.34 (0.17, 0.70)
Immunisation status	Complete	6 (37.5)	133 (44.6)		
	Incomplete	10 (62.5)	165 (55.4)	1.34 (0.48, 3.79)	0.94 (0.30, 2.93)
Presence of comorbidity	No	1 (6.2)	19 (6.4)		
	Yes	15 (93.8)	279 (93.6)	1.02 (0.13, 8.15)	0.81 (0.87, 7.55)
Gender	Female	7 (43.8)	141 (47.3)		
	Male	9 (56.3)	157 (52.7)	1.16 (0.42, 3.18)	1.16 (0.40, 3.33)
Any severe malnutrition (WAZ or WHZ z-score<−3, MUAC<11.5 cm)	No	12 (75.0)	274 (92.0)		
	Yes	4 (25.0)	24 (8.1)	2.17 (0.79, 5.96)	4.08 (0.08, 7.55)

aOR – adjusted odds ratio, CI – confidence interval, cOR – crude odds ratio, MUAC – mid-upper-arm circumference, SD – standard deviation WAZ – weight for age Z score, WHZ – weight for height Z score

*Values are means ± SDs or n (%).

## DISCUSSION

All children enrolled in our study survived. A tiny proportion was reported by caregivers to be worse or to have had no change in clinical status compared with baseline. Health workers followed IMCI guidance and treated all enrolled children with lower chest indrawing pneumonia with oral amoxicillin on an outpatient basis. Most mothers also adhered to the prescription advice. Only two children were lost to follow-up.

Our survival data are comparable to those of two observational studies: one conducted at an outpatient level in Papua New Guinea [34] and another across four countries (Bangladesh, Egypt, Ghana, and Vietnam) [35]. However, a community-based observational study in Kenya reported five deaths out of 1906 children with indrawing pneumonia [36]. Community-based randomised controlled trials (RCTs) conducted elsewhere reported a case-fatality rate (CFR) of less than 1% among children aged 2–59 months with chest indrawing pneumonia treated at home with oral amoxicillin [37–39]. Hospital-based RCTs in which one group received oral amoxicillin at home also reported a CFR of less than 1% [16,18,40,41]. However, a retrospective analysis from Kenya [26] reported a CFR of 2.7% in children with pneumonia hospitalised in several district hospitals. This difference from our study is probably because our study was conducted at primary health care levels, which are less likely than hospitals to receive very sick patients. Our results, therefore, suggest that children with lower chest wall indrawing without any danger signs can be successfully treated with oral amoxicillin on an outpatient basis, in line with the WHO recommendations [20,21].

The high rates of correct prescription, especially in infants, of adherence to oral amoxicillin and clinical cure in our study indicate that this approach to managing chest indrawing pneumonia among children was a successful implementation of IMCI. The IMCI refresher training provided to health workers also contributed to these results. Several post-training follow-up studies have reported similar results among primary health care workers who correctly follow IMCI protocols [42–44]. Still others have emphasised the benefits of frequent, regular follow-up by supervisors and the importance of ensuring the availability of requisite commodities [42,45].

A small proportion (5%) of children were admitted to a hospital during the follow-up period; most had recovered and were discharged by day 15. Children aged 12–59 months were hospitalised more often than younger children. Along with comorbidities, children who were hospitalised might have received inadequate amounts of oral amoxicillin. Those who adhered to the appropriate treatment duration were not hospitalised. Children with any form of malnutrition were more likely to be hospitalised compared to children with normal growth. Our hospitalisation rate is slightly higher than the 4.3% reported by Morre et al. in an outpatient-based observational study in Papua New Guinea [34] and the 3.9% were hospitalised in an outpatient-based observational study from four countries [35]. Our hospitalisation rate is similar to the 5.4% reported in an RCT in India among children randomised to the home-based oral amoxicillin arm [40], and higher than the 1.7% rate reported in the home oral amoxicillin group in another RCT in Pakistan [46].

In our study, however, a large proportion of children who were hospitalised had comorbidities, including malaria and diarrhoea. Onono et al. from Kenya reported an association of comorbidities such as malaria and diarrhoea with treatment failure [36]. A large descriptive study from Uganda reported that over one-third of under-five-year-old patients presenting with multiple diagnoses/comorbidities, with the overlap most common among patients with malaria, cough/cold, pneumonia, diarrhoea, and intestinal infections [47]. Trained health workers should use IMCI to assess children not only for pneumonia but also for other comorbidities, and to manage these children according to IMCI protocols.

Our results on the successful management of chest indrawing pneumonia without danger signs among children 2–59 months on an outpatient basis with oral amoxicillin have important public health policy and programmatic implications in Uganda. First, the cost of outpatient treatment is much lower than that of a hospital referral and admission. In a systematic cost analysis, the cost of pneumonia treatment was reported as USD 51.7 at an outpatient facility compared with USD 242.7 at hospitals[48]. 'Cost' is calculated for both the health system and families. One study from India reported that the direct out-of-pocket cost of hospitalised ARI was 34% of annual per capita income [49]. Most household expenditures were for medicines, food, hospitalisation, transport and loss of wages [48,50,51]. Second, the lower number of hospitalisations reduces the risk of health care-associated infections (HAIs), which is a significant problem in many countries [52,53]. The HAIs can result in substantial morbidity and mortality in hospitals [54,55]. Third, increased use of broad-spectrum antibiotics results in antimicrobial resistance (AMR). Bacterial infections cause an estimated 7.7 million deaths, of which 1.3 million are by antimicrobial-resistant bacteria [56,57]. Standard case management of pneumonia, as recommended by IMCI, not only reduces injectable antimicrobial use [58] but also saves lives [59,60]. Therefore, ambulatory treatment of childhood pneumonia is not only cost-effective but also saves money for both families and the health system, while reducing the risk of HAI and AMR by reducing hospitalisations.

Because the study setting and populations are similar across the country, this practice could be implemented nationwide. It will require IMCI-trained health workers with access to appropriate commodities. Outpatient management of chest indrawing pneumonia in children has the potential to improve access to care and decrease inequities, while reducing costs of care and mortality from pneumonia. Caregivers should be advised on when to seek additional care if the child's condition worsens. Caregivers also should be linked to community-level health workers to enable enhanced follow-up and health-seeking behaviour.

Our study had several strengths. We had a reasonable sample size to establish, with optimal precision, the estimate of survival in children with chest indrawing pneumonia in the district. Participating health facilities were distributed across the district's varying administrative units based on the patient load they served, with variations in care for children and in caregivers’ health-seeking behaviour during follow-up. Our study was conducted in a programme setting with a heterogeneous group of children having varying nutritional status. We had a well-established system of participant follow-up at the community level to identify and refer any child who became sicker, and caregivers were appropriately counselled on when to seek further care.

Nonetheless, our study was not without limitations. The study’s outcomes, regarding the child’s condition on day 15, were assessed based on maternal report, introducing subjectivity. However, the refresher IMCI training for health providers to provide quality care and the prospective follow-up of individual children to assess survival status, strengthens confidence in our study results. Adherence to prescribed treatment was not objectively assessed; it was based on caregivers' self-reported information, which might have under- or overestimated adherence levels. Oxygen saturation was not assessed using pulse oximetry to identify children who might have needed hospital admissions. Regardless of the level of adherence and level of correct prescription aligned to IMCI, survival was 100% even among children who were hospitalised.

## CONCLUSIONS

This study indicates that children with chest indrawing pneumonia without danger signs can be effectively and safely managed at home with oral amoxicillin for five days. It means that the child is identified at the health facility by trained health workers and that appropriate commodities are available. Our results also indicate that follow-up after the treatment initiation, as recommended by IMCI (day 3), is vital for the outpatient management of chest indrawing pneumonia, as most children who needed hospitalisation did so within 4 days. There is a need to emphasise the importance of adequate counselling on when to seek further care and to link with community health workers for better follow-up. Our results support the IMCI policy and programmatic approach of treating chest indrawing pneumonia in outpatient settings. This approach could improve access to health care, reduce hospital referrals and admissions, and lower health care costs, thereby ultimately improving equity for vulnerable populations.

## Additional material


Online Supplementary Document

**Figure 1. **Study participant profile. RTI – respiratory tract infection.
