# Supplementary material for: Treatment practices and outcomes of chest indrawing pneumonia in children aged 2–59 months in primary health facilities of Kamuli District, Eastern Uganda
Source: J Glob Health. 2026 Jan 23;16:04021. doi: 10.7189/jogh.16.04021 (PMC12828440; doi:10.7189/jogh.16.04021)
Supplement: Online Supplementary Document [file jogh-16-04021-s001.pdf]

Supplement to: Mupere E, Nasasira M, Tabwenda L, Babikako HM, Muhirwe L, Nsungwa-Sabiiti J, Qazi SA, Nisar YB. Treatment practices and outcomes of chest indrawing pneumonia in children aged 2–59 months in primary health facilities of Kamuli District, Eastern Uganda. J Glob Health. 2026;16:04021.

Table S1. Co-morbidity classified using the IMCI algorithm (n = 296)

**Characteristics**	**n (%)**
Any comorbidity	296
One comorbidity	225 (76.0)
Two or more comorbidities	71 (24.0)
Comorbidity	
Confirmed malaria infection*	170 (53.8)
Ear infection	1 (0.3)
Palmar pallor	3 (1.0)
Diarrhoea	63 (20.1)
Measles	17 (5.4)
Moderate Acute Malnutrition as assessed by the health worker	9 (2.9)

* Malaria diagnosis was ascertained using a malaria rapid diagnostic test

Table S2. Management practices for patients who were followed up (n=314)*

Variable		n (%)
The health worker prescribed the correct dose of oral amoxicillin†		136 (43.3)
Correct dose prescribed by age†	2-11 months– 250mg	93/136 (68.4)
	12-59 months– 500mg	43/136 (31.6)
Adherence to the prescribed oral amoxicillin at home (n=312) ‡	10 doses (five days)	202 (64.7)
	6 to 9 doses (3 to 4 days)	104 (33.3)
	<6 doses (less than 3 days)	6 (1.9)

* Two children were lost to follow-up
†Correct dose was considered to be the IMCI recommended age-specific amoxicillin dose, twice daily for five days [20,21].
‡ Two children had missing data on the adherence to the prescribed oral amoxicillin
§ Hospitalisation was defined as any admission at any time between day 2 and day 14.
